# The Effect of SMA Fiber Content on the Bending and Self-Recovery Performance of ECC Beams

**DOI:** 10.3390/ma16155319

**Published:** 2023-07-28

**Authors:** Zhao Yang, Yun Ren, Qing Wu

**Affiliations:** 1 School of Urban Construction, Wuhan University of Science and Technology, Wuhan 430065, China; yrenlyfy@163.com (Y.R.); qinng28@163.com (Q.W.); 2 Hubei Provincial Engineering Research Center of Urban Regeneration, Wuhan University of Science and Technology, Wuhan 430065, China

**Keywords:** superelastic SMA fibers, ECC, bending strength, self-recovery, energy dissipation

## Abstract

The addition of superelastic shape memory alloy fibers (SMAF) into engineering cementitious composites (ECC) can create a new type of SMAF-ECC composite material with good self-recovery and energy dissipation performance, which is very suitable for seismic structures. In this study, 10 groups of beam specimens with different volume contents of SMAF were fabricated, and the bending performance, deflection recovery and energy dissipation ability of these beams were studied through three-point bending cyclic loading tests. The failure mode, peak load, load–deflection curve, crack width and other indicators of the specimens were analyzed, and the relationship expression between fiber content and bending strength was established by fitting analysis. The results show that adding SMA fibers can significantly improve the peak load of ECC beams, with a maximum increase of 48.31%. The knotted SMA fibers can fully exert their superelasticity, providing the beam specimens with crack self-closing and deflection recovery ability. When the volume content of SMA fibers is 0–0.6%, the bending strength, energy dissipation ability and deflection recovery ability of the composite material beams increase with the increase in fiber content. When the volume content of SMA fibers is 0.6–1.0%, the above indicators decrease with the increase in fiber content. The suggested equations can well reflect the relationship between fiber content and beam bending strength. The research results of this paper provide theoretical support for the engineering application of SMAF-ECC composite materials.

## 1. Introduction

Since ancient times, seismic disasters have been one of the most destructive natural disasters faced by humans. Taking China as an example, over 70% of its cities and more than 50% of its population are located in areas prone to earthquakes [1]. How to effectively improve the seismic resistance and post-earthquake reparability of buildings is a pressing issue that needs to be addressed [2]. In recent years, many scholars have begun to study “self-healing” structures, and the use of high-performance materials to achieve the self-healing properties of structures is the most simple and feasible method.

Shape memory alloys (SMAs) have unique shape memory effects and superelasticity and are widely used in civil engineering, mechanical engineering, biomedical engineering, and other fields [3,4]. When superelastic SMAs are used in civil engineering structures, they can provide excellent self-recovery and energy dissipation capabilities to structures, effectively reducing residual deformation of structures. M.A. Youssef [5,6] and others conducted experimental studies on the seismic performance of reinforced beam-column joints using superelastic shape memory alloy, replacing ordinary longitudinal steel bars with SMA rods. The results showed that the structure had good self-repositioning and energy dissipation capabilities. Saiidi and Wang [7] used SMA bars instead of ordinary steel bars in concrete columns to explore the use of superelastic SMAs to recover plastic deformation after earthquakes. The experimental results showed that the hysteresis curve of the column under cyclic loading presented an obvious flag shape, and the residual deformation of the column was very small. Nailiang Xiang [8] analyzed the seismic performance of concrete piers with superelastic SMA bars and found that SMA bars can not only improve the self-recovery performance of concrete piers but also increase the ultimate lateral displacement of the piers, reducing the overall energy dissipation. The adoption of SMA reinforcement in the plastic hinged area of the piers significantly reduces the seismic vulnerability of the bridge. However, when SMA is directly applied to ordinary concrete structures, the excellent performance of SMA materials cannot be fully realized due to the non-coordination of deformation between SMA and concrete and the brittle failure of concrete under earthquake action.

Engineering cementitious composites (ECC) are composite materials composed of cement, fine sand, and other admixtures, and are reinforced with randomly dispersed short-cut fibers such as polyvinyl alcohol (PVA) or polyethylene (PE) to enhance toughness [9]. Typically, when the tensile strain reaches 3–5%, the crack spacing is 3–6 mm, and the crack width is approximately 60 μm [10]. This can effectively solve the cracking problem of concrete, and the ductility of ECC is far superior to that of ordinary concrete, significantly improving the seismic performance and deformation capacity of concrete structures. However, the high toughness and multi-cracking ductility characteristics of ECC come at the cost of significant residual deformation and extensive microcracking damage [11], which is still unfavorable for post-earthquake repair and functional recovery of structures. Therefore, combining SMA with ECC can achieve high ductility of ECC while utilizing the deformation recovery force of SMA to solve the residual deformation problem of ECC structures after earthquakes.

Hung [12] investigated the bending behavior of SMA-reinforced ECC members and steel-reinforced ECC beam members. Steel-reinforced cantilever beams and SMA-reinforced cantilever beams were fabricated and tested under cyclic loading. The study found that conventional steel-reinforced ECC members were prone to fracture, and the steel reinforcement was susceptible to debonding in the plastic hinge zone. In contrast, SMA-reinforced ECC members exhibited improved ductility and excellent energy dissipation and self-healing properties due to the inclusion of superelastic SMA reinforcement. F. Hosseini et al. [13] proposed a novel Cu-Al-Mn SMA-ECC bridge pier, which combines the high energy dissipation of ECC and the superelasticity of SMA to enhance the seismic performance of bridge piers. Experimental results showed that the use of ECC material can reduce the damage deformation of components under seismic loads compared to traditional RC components, and the incorporation of SMA-ECC can reduce the residual deformation of the bridge pier by 90%. Ge J et al. [14] developed a three-dimensional computational model using the finite element software OpenSees through a computational study of the seismic response of a three-span highway bridge system, analyzing and comparing two versions of the same bridge, one with conventional cast-in-place reinforced concrete columns and the other with SMA reinforcement and ECC top plastic hinges. It was found that the new SMA/ECC plastic hinges significantly reduced the damage and post-earthquake residual displacements of the bridge substructure and improved the post-earthquake suitability of the bridge after a strong earthquake. Amirmozafar Benshams [15] found that SMA reinforcement improves the lateral anti-collapse capacity of bridge piers, enhances their self-recovery ability, reduces the residual drift at the end of loading cycles, and prevents the accumulation of plastic deformation in continuous loading cycles. The above studies mainly used continuous materials such as SMA bars, which have inherent drawbacks such as high cost, difficulty in connecting with steel reinforcement, and potential defects.

Compared with SMA bars, SMA fibers (SMAF) have fewer inherent defects and better material properties, are easy to manufacture, and have lower production costs. In particular, the uniform distribution of fibers is more suitable for the large-scale cracking of ECC [16]. Therefore, the introduction of superelastic SMAF into high-ductility ECC to form a new type of SMAF-ECC composite material can fully leverage the excellent properties of both materials. SMA fibers can fully exert their superelastic properties, providing recovery force for ECC components after earthquakes, such as closing cracks and restoring deformation. At the same time, the high ductility of ECC can achieve better deformation coordination with SMA fibers, and the cracks in ECC are fine and dense, making it easy for SMA fibers to close cracks. Song et al. [17] found that the compressive, flexural and bending strengths of SMAF-ECC specimens were significantly improved compared with those of the control group without SMA fibers, and that the specimens had obvious strain-hardening behaviors and crack self-closing properties through four-point bending tests on ECC specimens doped with SMA fibers. Weihong Chen et al. [18] evaluated the crack self-healing ability of SMA fiber-reinforced ECC. The results of ultrasonic pulse testing and bending tests were consistent, showing that compared with concrete specimens, specimens with SMA-ECC had significantly fewer cracks and smaller crack widths, indicating that SMA-ECC had good crack healing ability and could be used to reduce structural damage and repair cracks. Zhao Yang et al. [19] showed that when SMA fibers are effectively anchored in the ECC matrix, the superelastic characteristics of SMA fibers can be fully utilized, providing a flag-shaped hysteretic energy dissipation capacity for SMAF-ECC beams and providing recovery force for composite material beams during unloading, enabling the beam to achieve self-closing of cracks and self-recovery of deflection. Meanwhile, Zhao Yang et al. [20] found that by setting up the end-knotted form of fibers, the SMAF-ECC half-dog bone specimens were subjected to direct drawing tests, and it was concluded that the knotted-end form could effectively improve the bonding performance between SMA fibers and ECC matrix, which provided the basic conditions for making full use of the superelasticity of SMA materials. In addition, Zhao Yang et al. [21] conducted uniaxial cyclic tensile tests to investigate the self-recovery performance of SMAF-ECC under cyclic tensile. Doping SMA fibers in ECC can increase the ultimate strain and ultimate tensile strength of the specimen, and significantly reduce the residual crack width and residual deformation of the specimen when unloaded, and the maximum strain recovery and crack recovery of the specimen obtained from the test reached The maximum strain recovery rate and crack recovery rate of the test specimens reached 69% and 77%, respectively, showing good crack closure and deformation recovery ability.

However, it must be noted that the current research on SMAF-ECC is still in its infancy, with a severe lack of relevant literature, and therefore a substantial amount of fundamental research is urgently needed to investigate the material’s mechanical properties. Previous studies have shown that the addition of SMA fibers significantly impacts the mechanical behavior of beams [22], highlighting the need for further research on the effects of SMA fiber content on the bending and self-recovery performance of ECC beams. This study employs a three-point bending test to analyze various mechanical performance indicators, such as peak load, bending strength, energy dissipation capacity, and self-recovery ability under deflection, to compare the impact of different volume percentages of SMA. The results of this research can provide a theoretical basis for the application of this new type of SMAF-ECC material in structural components.

## 2. Experiment Materials

### 2.1. Superelastic SMA Wire

**Figure 1:**
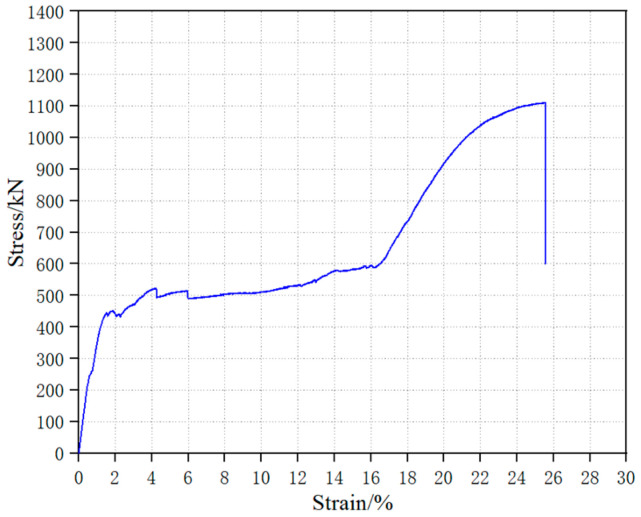
Uniaxial tensile curve of 1.0 mm superelastic SMA.

**Figure 2:**
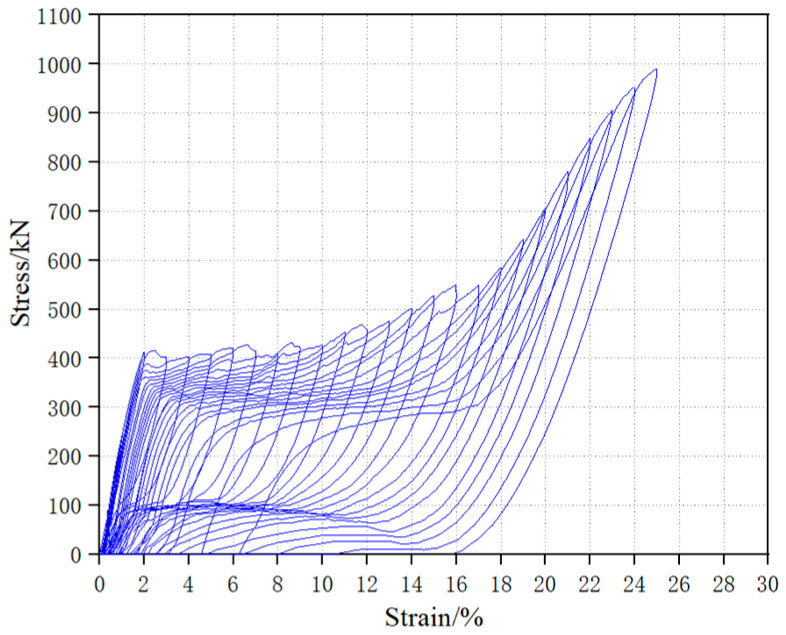
Cyclic tensile curve of 1.0 mm superelastic SMA.

In the experiment, a short-fibered superelastic SMA wire with a diameter of 1.0 mm was produced, with a Ni-Ti ratio of 55.86% and 44.14%, respectively. The SMA wire was subjected to tensile tests according to AASHTO T 68M/T68-09[S] [23]. Prior to the tensile test, the SMA wire was subjected to cold and hot treatments following the method described in the literature [19] to stabilize its mechanical properties. The length of the SMA wire for the tensile test was 100 mm, and the temperature in the laboratory was maintained at 25 °C. The displacement control loading method was adopted with a constant displacement rate of 0.02 mm/s throughout the loading process [24]. The uniaxial tensile stress–strain curve is shown in Figure 1, and the cyclic tensile stress–strain curve is shown in Figure 2.

As shown in Figure 1, the starting stress of the phase transformation plateau of the SMA wire was 441.86 MPa, with a corresponding strain of 1.55%, and the ending stress was 589.63 MPa, with a corresponding strain of 16.29%. The ultimate tensile strength was 1111.01 MPa, with a corresponding ultimate strain of 25.53%. Figure 2 shows that within the first 12 cycles (with a tensile strain ≤ 12%), the stress–strain curve of the SMA wire exhibited an obvious flag shape, and the unloading residual strain was within 1%. Experimental data indicate that the SMA material possesses excellent superelastic performance at room temperature.

### 2.2. ECC

**Figure 3:**
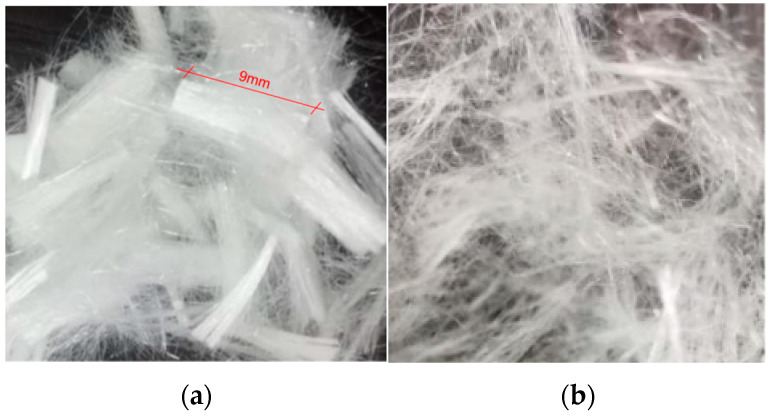
PVA fiber before and after treatment. (**a**) PVA fiber before treatment; (**b**) PVA fiber after treatment.

The engineered cementitious composites were prepared using the mixture proportion previously used in the research of the project team [19], as shown in Table 1. The raw materials mainly included P.II 42.5 grade Portland cement, Class I fly ash, 100–200 mesh quartz sand, 540P polycarboxylate superplasticizer, and 9 mm chopped PVA fibers. The PVA fibers were produced by Kuraray China Co., Ltd. in Shanghai, China, and their specific performance parameters are shown in Table 2. To ensure the uniform dispersion of PVA fibers in the matrix, they were subjected to a dispersion treatment, as shown in Figure 3. In addition, the PVA fibers were added to the mixing tank in batches during the stirring process to ensure their even distribution.

**Figure 4:**
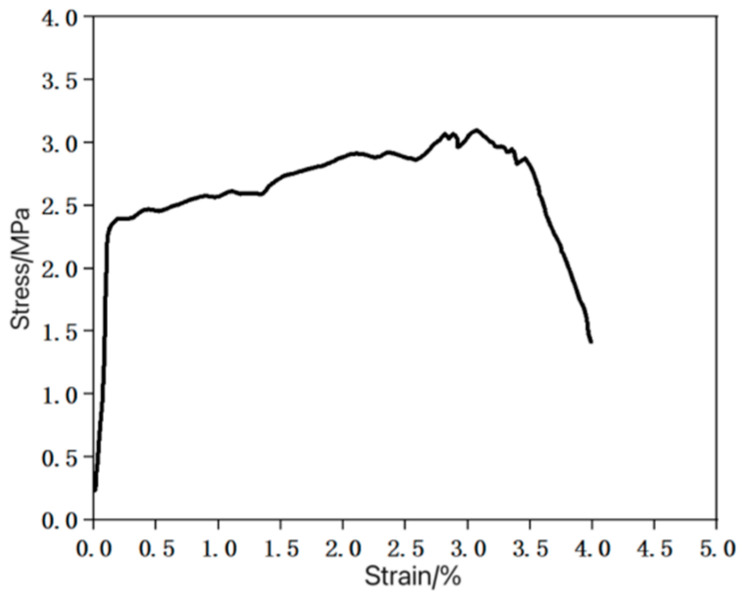
Stress–strain tensile curve of thin plate specimen.

**Figure 5:**
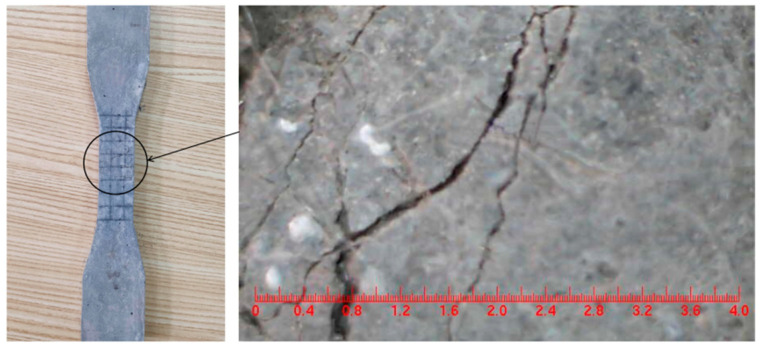
Tensile cracking diagram of plate specimen.

Dumbbell-shaped thin plate specimens were made according to the mix proportion in Table 1, in accordance with the Chinese standard JC/T2461-2018 [25], and uniaxial tensile tests were carried out. The stress–strain curve obtained from the test is shown in Figure 4. It can be seen from Figure 4 that the specimen exhibited obvious strain hardening and multiple cracking characteristics during the tensile test (see Figure 5), and the ultimate tensile strain reached more than 3%, which met the requirements of this test.

## 3. Test Design

### 3.1. Specimen Design

**Figure 6:**
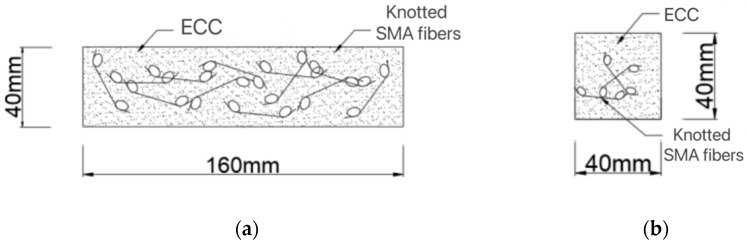
Composite beam diagram. (**a**) Elevation view; (**b**) Cutaway view.

**Figure 7:**
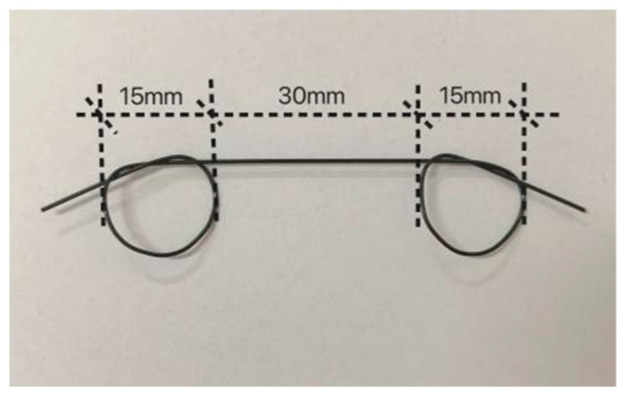
Knotted SMA fibers.

To investigate the influence of SMA fiber content on the bending and self-recovery performance of ECC beams, SMAF-ECC beam specimens were produced according to ASTM C348 [26] with dimensions of 40 × 40 × 160 (mm), as shown in Figure 6. The ECC was prepared according to the mix proportion shown in Table 1. The SMA fibers were made using the SMA wire in Section 2.1, with a diameter of 1.0 mm. According to the previous research results of the research group [19,27], the knotted end of the SMA fibers can significantly improve the bond strength between the SMA fibers and the ECC matrix, thus effectively exerting the superelasticity of the SMA material. Therefore, the knotted SMA fibers were used in this experiment. The straight-line distance between the two knots of the knotted fiber was 30 mm, and the knot diameter was 15 mm, as shown in Figure 7. Ten sets of beam specimens were designed according to different SMA fiber contents, among which nine sets were SMAF-ECC beams with SMA fibers and 1 set was an ECC beam without SMA fibers as a control specimen. Three specimens were produced for each group of beams, and the specific beam specimen design is shown in Table 3.

**Figure 8:**
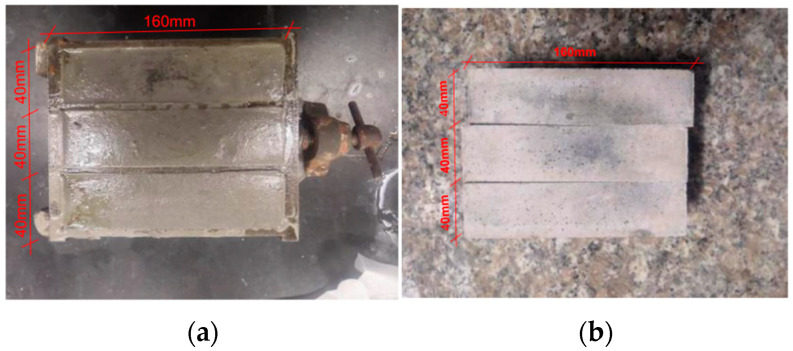
Specimen curing drawing. (**a**) Specimen curing for 24 h; (**b**) specimen curing for 28 days.

The SMA fiber with knot-shaped ends was added to the mixing bucket, and stirred clockwise manually using a stainless steel stirrer. After thorough mixing and homogenization, the mixed material was slowly poured into an oiled mold, as shown in Figure 8a, until it slightly exceeded the top of the mold. The mold was then placed on a vibrating table and vibrated for three cycles, with each cycle consisting of 60 vibrations. After vibration, the excess mixed material was scraped off with a trowel and the surface was smoothed, then the mold was placed in a curing box (temperature controlled at 20 ± 2 °C and relative humidity above 95%) for 24 h. The specimens were then demolded and numbered, as shown in Table 3. Finally, the specimens were placed in a curing box for 28 days, as shown in Figure 8b, and then subjected to mechanical performance tests.

### 3.2. Test Loading and Measurement

**Figure 9:**
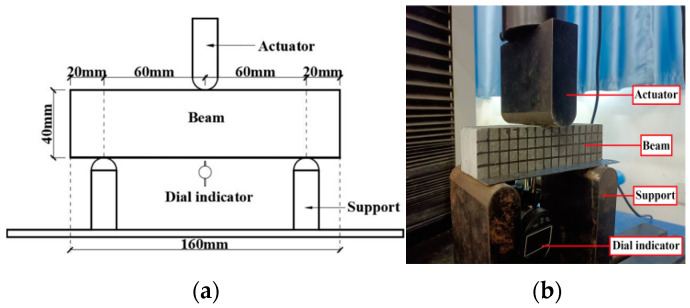
Schematic of three-point test loading. (**a**) Schematic loading diagram; (**b**) physical loading diagram.

**Figure 10:**
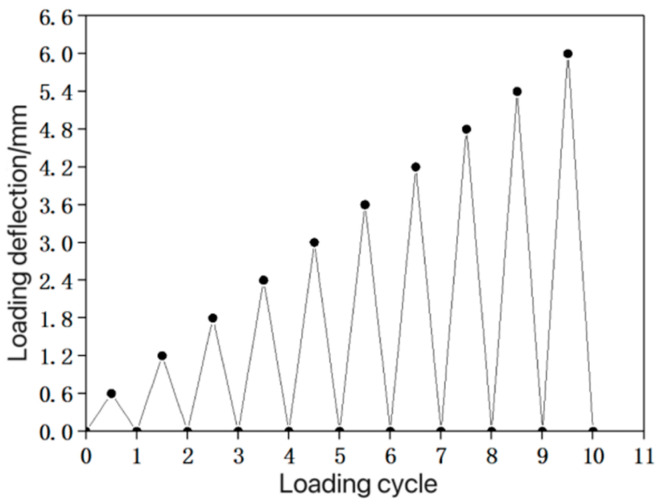
Test loading system.

Following the experimental method described in reference [28], three-point bending tests were conducted on composite material beams under cyclic loading. The loading point was located at the top of the mid-span section of the beam specimen, with an axial distance of 120.0 mm from the bottom two support points. A WD-P6305 universal testing machine was used to apply vertical cyclic loads to the beam. The displacement control method was employed during loading, with a loading rate of 0.6 mm/min. The cyclic loading consisted of 10 levels, with each stage having a cycle load of (1/10)Δ, where Δ represents the mid-span deflection of the control specimen at the ultimate load. In this test, Δ was 6 mm, and the loading device is shown in Figure 9, while the loading system is illustrated in Figure 10.

**Figure 11:**
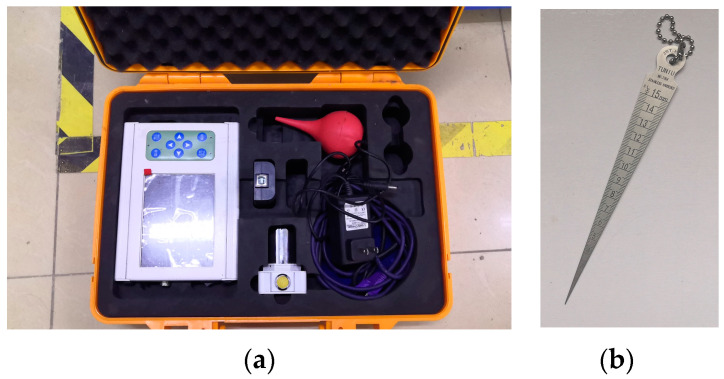
Crack width measuring equipment. (**a**) Intelligent crack width measuring instrument; (**b**) clearance scale.

The experimental load was measured by the load sensor integrated in the testing machine, while the mid-span deflection of the beam specimens was measured by a dial gauge located at the bottom of the beams, as shown in Figure 9. The crack width of the specimens was measured using an intelligent crack width measuring instrument and a gap gauge, as shown in Figure 11a,b, respectively.

## 4. Test Results

### 4.1. Control Specimen

**Figure 12:**
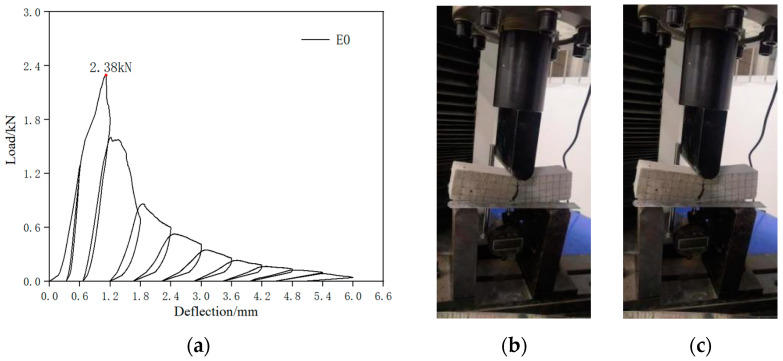
Contrast test cyclic loading. (**a**) Load–deflection curve; (**b**) maximum loading crack; (**c**) unload the residual crack.

The control specimen was an ECC beam without SMA fibers. The load–deflection curve and crack pattern of the control specimen were obtained by cyclic loading tests, as shown in Figure 12. As shown in Figure 12a, the peak load of the specimen was 2.38 kN, and no flag-shaped characteristic was observed in the load–deflection curve of the control specimen during the entire cyclic loading test. In the first two loading cycles, the loading deflection was relatively small, and the PVA fibers at the crack location played a bridging role, resulting in small crack widths. From the third loading cycle, a wide main crack appeared at the weak section of the specimen beam, where the PVA fibers at the crack location were broken or detached and unable to play a bridging role, resulting in a decrease in the bearing capacity of the specimen. When loaded to the tenth cycle, the residual deflection of the specimen was 5.01 mm, and the maximum crack width of the specimen reached 6.9 mm (Figure 12b). The residual crack width did not change significantly after unloading (Figure 12c).

### 4.2. SMAF-ECC Beam Specimen

**Figure 13:**
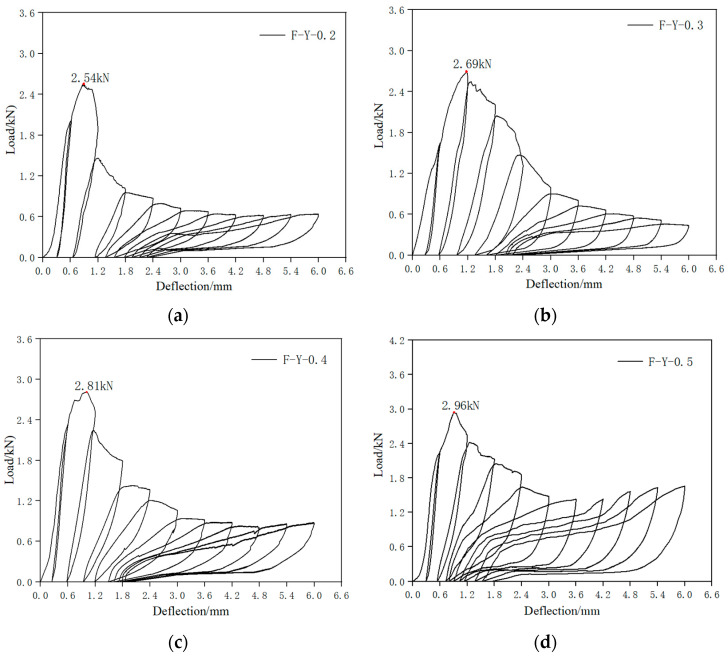

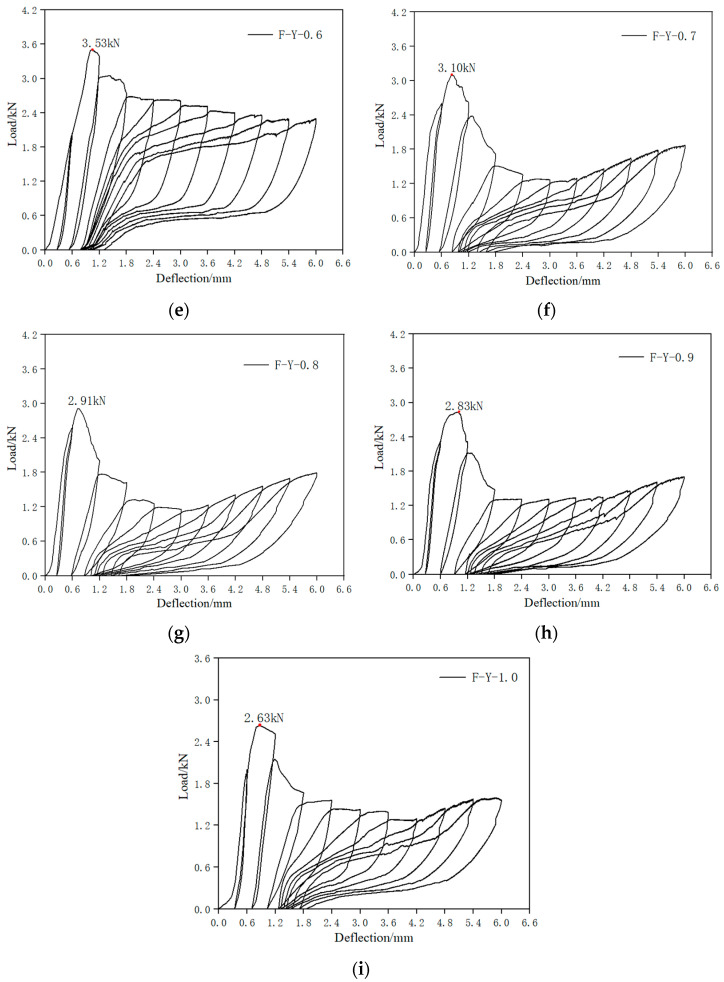
Load deflection curve of SMAF-ECC beam specimen. (**a**) F-Y-0.2 specimen; (**b**) F-Y-0.3 specimen; (**c**) F-Y-0.4 specimen; (**d**) F-Y-0.5 specimen; (**e**) F-Y-0.6 specimen; (**f**) F-Y-0.7 specimen; (**g**) F-Y-0.8 specimen; (**h**) F-Y-0.9 specimen; (**i**) F-Y-1.0 specimen.

**Figure 14:**
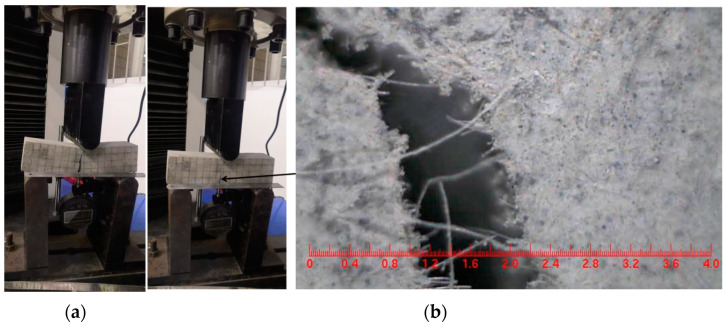
Crack diagram of F-Y-0.6 specimen. (**a**) Maximum loading crack of F-Y-0.6; (**b**) final unloading residual crack of F-Y-0.6.

The load–deflection curves of the SMAF-ECC beam specimens and the corresponding peak loads and crack widths are shown in Figure 13 and Table 4, respectively. As seen from Figure 14, all SMAF-ECC beam specimens exhibit clear flag-shaped load–deflection curves, indicating significant self-recovery ability after reaching a certain deflection. As shown in Table 4, the peak loads of the SMAF-ECC beam specimens are significantly higher than that of the control specimen, with specimen F-Y-0.6 exhibiting the largest peak load of 3.53 kN, which is a 48.31% increase compared to the control specimen. In the following analysis, specimen F-Y-0.6 will be taken as an example to investigate the experimental phenomenon of SMA fiber-reinforced specimens.

In the first four cycles, specimen F-Y-0.6 did not exhibit flag-shaped characteristics and had a large residual deflection after unloading. The absence of flag-shaped characteristics was due to the fact that the stress of the SMA fibers was small in the initial loading stage and did not reach the transformation platform stress, so the superelasticity could not be fully utilized. The large residual deflection was due to the initial slip of the SMA fibers before the anchoring effect was fully utilized at the fiber ends [29]. Starting from the sixth cycle, the load–deflection curve of the specimen showed flag-shaped characteristics, and the residual deflection decreased after unloading. The peak load of the specimen decreased slightly in the subsequent loading cycles, and the residual crack width decreased significantly. When loaded to the tenth cycle, the maximum crack width of the specimen reached 5.7 mm (Figure 14a), while the residual crack width after unloading was 1.2 mm (Figure 14b). This indicates that the stress of the SMA fibers had exceeded the starting stress of the transformation platform, and the superelasticity of the SMA had been effectively utilized. During loading, the SMA fibers could effectively bear the load and help the specimen exhibit flag-shaped energy dissipation characteristics. During unloading, the SMA fibers could provide significant recovery force to the specimen, effectively reducing the residual deflection and residual crack width [22].

### 4.3. Impact Analysis of SMA Fiber Content

#### 4.3.1. Bending Strength

According to the standard test methods for fiber-reinforced concrete CECS 13:2009 [30] and ASTM C 1609 [31], the bending strength can be calculated according to Equation (1):

(1) $$f=\frac{PL}{bh^{2}}$$

where *f* represents the bending strength in MPa, *P* represents the applied load in N, *L* represents the span length in mm, *b* represents the width of the cross section in mm, and *h* represents the height of the cross section in mm. Based on Equation (1), the bending strengths of each specimen can be calculated and are shown in Table 5.

Based on Table 5, it can be seen that the bending strength of the specimens containing SMA fibers is higher than that of the specimens without fibers. This indicates that randomly distributed SMA fibers can significantly improve the bending strength of the ECC matrix. When the volume content of SMA fibers is 0.6%, the bending strength of the specimens is the highest, reaching 6.61 MPa, which is 48.2% higher than that of the control specimen.

When the volume content of SMA fibers is between 0% and 0.6%, the bending strength of the specimens increases with the increase in the volume content of SMA fibers. For every 0.1% increase in the volume content of SMA fibers, the bending strength of the specimens increases by approximately 0.36 MPa. When the volume content of fibers is low, the SMA fibers are dispersed in the ECC matrix, and the fiber reinforcement effect is limited. As the fiber content increases, more SMA fibers participate in the stress transfer, control crack development through bridging effect, and effectively improve the bending strength of the specimens.

However, when the volume content of SMA fibers is between 0.6% and 1.0%, the bending strength of the specimens decreases with the increase in the volume content of SMA fibers. For every 0.1% increase in the volume content of SMA fibers, the bending strength of the specimens decreases by approximately 0.42 MPa. This is because when the fiber content is too high, Van der Waals forces exist between the fibers [32], which easily causes fiber clustering during the mixing process, increases the air content in the composite material, and weakens the bonding ability between SMA fibers and ECC interface. At the same time, the clustering also causes uneven distribution of SMA fibers, which makes it easier for SMA fibers to detach from the matrix at local sites such as cracks, resulting in premature pulling out of SMA fibers. Both cases lead to a decrease in the bending strength of the specimens. These conclusions are similar to the research findings of Eunsoo Choi [33].

**Figure 15:**
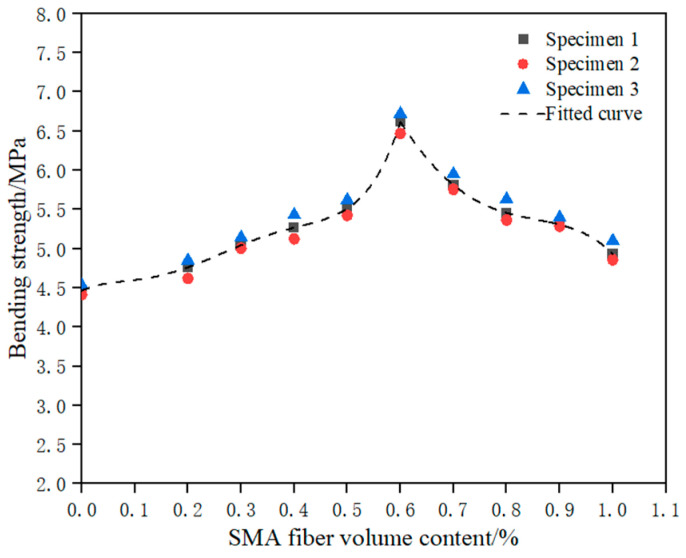
The fitting curve of SMA fiber volume content and bending strength.

As can be seen from the above, the SMA fiber content has a significant impact on the bending strength of the specimens. To quantitatively describe this influence, the fiber content influence coefficient *k_v_* for bending strength is introduced. Let the bending strength of specimens with an SMA fiber volume content of 0.6% be the base value *f*_0_. Then, the bending strength *f* of specimens with any fiber content can be expressed as Equation (2):

*f = k × f_0_* (2)

where the value of *k* is determined by the fiber content. Then, the mathematical relationship between *k* and the fiber content *V* is established through data fitting. The specific steps are as follows: First, based on the data from Table 5, the ratio of the bending strength of specimens with other fiber contents to the base value *f*_0_ is obtained, thus obtaining the *k_v_* value for each specimen. Then, the SMA fiber content is divided into two intervals: 0–0.6% and 0.6–1.0%. Using Origin software, separate curve fitting is performed for *k_v_* and *V* in each interval, as shown in Figure 15, and the expressions for the relationship between *k_v_* and *V* are obtained as shown in Equations (3) and (4). In Figure 15, specimens 1, 2, and 3 represent the three specimens in each group. It can be observed from Figure 15 that the fitted curves align well with the experimental data, indicating that the proposed calculation formula can accurately describe the influence of fiber content on bending strength.

*k_v_* = 0.67613 + 0.49498*V* − 5.65352*V*^2^ + 35.41824*V*^3^ − 82.34052*V*^4^ + 65.39957*V*^5^ (0 ≤ *V* < 0.6%) (3)

*k_v_* = 1.65374 + 4.88369*V* − 23.28385*V*^2^ + 29.31165*V*^3^ − 11.81921*V*^4^ (0.6% ≤ *V* ≤ 1%) (4)

#### 4.3.2. Energy Dissipation Capacity

**Figure 16:**
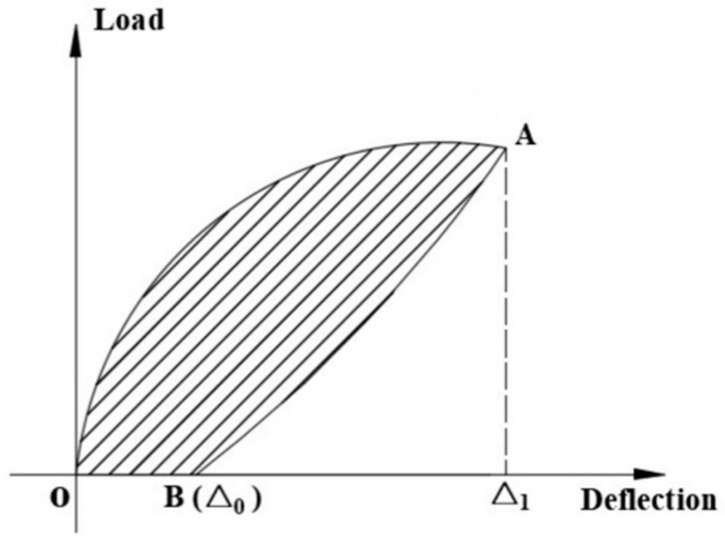
Schematic diagram of energy consumption calculation.

Energy dissipation refers to the energy absorbed by a specimen through a certain amount of deformation during cyclic loading and unloading. The energy dissipation can be calculated by the area enclosed by the load–displacement hysteresis loop for each cycle (as shown in Figure 16), and the formula for calculating energy dissipation is given in Equation (5) [34].

(5) $$E_{S}=S_{OAB}$$

where *E_S_* represents the energy dissipation in a single cycle, and *S_OAB_* is the area enclosed by the load–deflection curve in a single cycle. In the figure, A is the point of maximum displacement under loading, and B is the point where the load returns to zero after unloading.

**Figure 17:**
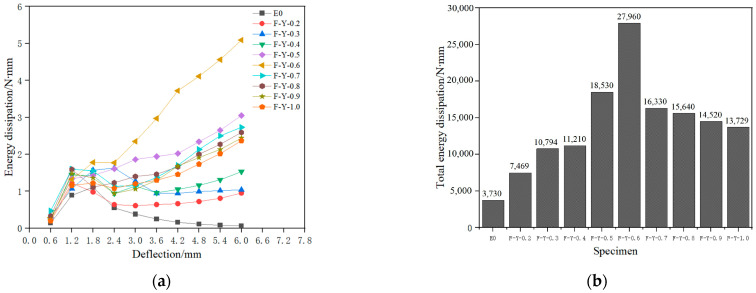
Comparison of Energy consumption. (**a**) Energy dissipation comparison chart of specimens; (**b**) comparison of total energy dissipation of specimens.

Based on Equation (5), the energy consumed by each specimen in each cycle was calculated. The energy consumption comparison of each specimen in each loading cycle is shown in Figure 17a, and the total energy consumption comparison of each specimen during the loading process is shown in Figure 17b.

**Figure 18:**
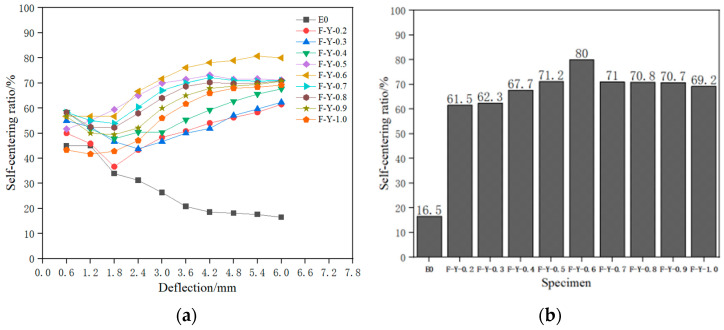
Comparison chart of deflection self-recovery. (**a**) The self-recovery rate of each stage of the load cycle; (**b**) load deflection of 6 mm self-recovery rate.

According to Figure 18a,b, it can be seen that the energy dissipation capacity of specimens with added SMA fibers is greater than that of the comparison specimens without fibers. Among them, the specimen with 0.6% volume content of SMA fibers has the strongest energy dissipation capacity, indicating that SMA fibers can be used to improve the energy dissipation capacity of ECC matrix by utilizing its unique hysteretic energy dissipation characteristics.

According to Figure 18a, the energy dissipation capacity of each specimen increases at the initial stage of loading. As the loading displacement increases, there are significant differences in the energy dissipation capacity among the specimens. Figure 18b shows that when the volume content of SMA fiber is between 0% and 0.6%, the energy dissipation capacity of the specimens increases with the increase in volume content. For every 0.1% increase in SMA fiber volume content, the total energy dissipation of the specimens increases by approximately 4038.33 N·mm. Among them, specimen F-Y-0.6 has the highest overall energy dissipation capacity, reaching 27,960 N·mm. This is because as the volume content of SMA fiber increases, more SMA fibers exhibit superelasticity and effectively enhance the energy dissipation capacity of the specimens by utilizing their flag-shaped hysteresis energy dissipation characteristics.

However, when the volume content of SMA fibers was between 0.6% and 1.0%, the energy dissipation capacity of the specimens decreased as the volume content increased. For every 0.1% increase in SMA fiber volume content, the total energy dissipation of the specimens decreased by approximately 3557.75 N·mm. This is because too many SMA fibers can form fiber clusters, leading to a decrease in bond strength and local fiber debonding, which reduces the overall energy dissipation capacity of the specimens. This is consistent with the analysis of bending strength in Section 4.3.1.

#### 4.3.3. Self-Recovery Ability of Deflection

To evaluate the effect of different SMA fiber contents on the self-recovery ability of ECC beams, Equation (6) [35] was used to calculate the deflection self-recovery rate γ of the specimens and evaluate the deflection self-recovery ability of the composite material beams. The deflection self-recovery rates of each specimen were compared, as shown in Figure 18. Table 6 records the average deflection self-recovery indicators of three specimens in each group in the last loading cycle (with a loading displacement of 6 mm). The standard deviation in the table was calculated based on the deflection self-recovery rates of the three specimens in each group.

(6) $$\gamma =\left(\gamma_{max}-\gamma_{residual}\right)/\gamma_{max}$$

where *γ*_max_ represents the maximum mid-span deflection of the beam specimen during loading in each cycle, while *γ*_residual_ residual represents the residual deflection of the beam specimen during unloading in each cycle.

According to Figure 18a,b, the deflection self-recovery ability of ECC beams containing superelastic SMA fibers was improved to varying degrees throughout the entire loading cycle compared to the control specimens. Among them, the F-Y-0.6 specimen had the best self-recovery effect, with a self-recovery rate of over 60% after the loading deflection reached 2.4 mm, and a recovery deflection of 4.68 mm and a self-recovery rate of 80% were achieved in the last loading cycle (loading deflection of 6 mm). This indicates that the knotted end can provide sufficient anchoring force for the SMA fibers, enabling them to effectively exhibit superelasticity and provide recovery ability for the deflection of the specimen.

When the volume content of SMA fibers is between 0% and 0.6%, the self-recovery ratio of the specimen increases with the increase in volume content, and the average self-recovery ratio of the specimen increases by approximately 7.15% for every 0.1% increase in SMA fiber content. Among them, the F-Y-0.6 specimen has the highest self-recovery ratio of 70.2%, which is due to the superelastic properties of SMA fibers providing recovery force and playing a “bridging effect” to heal cracks and increase the self-recovery of the specimen’s deflection. However, when the volume content of SMA fibers is between 0.6% and 1.0%, the self-recovery ratio of the specimen decreases with the increase in volume content, and the average self-recovery ratio of the specimen decreases by approximately 3.45% for every 0.1% increase in SMA fiber content. This is because at this point, the fiber content is too high, resulting in fiber clustering, which reduces the gripping force between the matrix and the superelastic SMA fibers, amplifies the defects within the matrix, and leads to a decrease in the self-recovery ratio of the specimen’s deflection, which is consistent with the reason for the decrease in energy dissipation capacity of the specimens with fiber volume contents of 0.6% to 1.0% in Section 4.3.2. This result is similar to the research results of Weihong Chen et al. [36].

## 5. Conclusions

This study investigates the effect of shape memory alloy (SMA) fiber content on the bending and self-recovery performance of engineered cementitious composites (ECC) beams. Three-point bending tests were conducted to analyze the load–deflection curve, bending strength, energy dissipation, and deflection recovery rate of the SMAF-ECC specimens, and the influence of SMA fiber content was studied. The main conclusions of this study are as follows:

(1) The addition of SMA fibers can improve the peak load of the ECC beam, up to 3.53 kN, which is a 48.31% increase compared to the control specimen without SMA fibers. The knotted SMA fibers can fully utilize their superelasticity to provide the flag-shaped hysteresis energy dissipation characteristic for the matrix, and provide significant restoring force to the specimen during unloading, effectively reducing the residual deflection and residual crack width, and achieving the functions of crack self-closure and deflection recovery for the specimen.

(2) When the SMA fiber content is less than 0.6%, with the content increasing, more SMA fibers participate in the bridging effect, resulting in an increase in the beam bending strength. However, when the content exceeds 0.6%, further increasing the content will cause fiber clustering, leading to a continuous decreasing in the bending strength. The highest bending strength in the test can be 48.2% higher than that of the control specimen. The suggested equations can well reflect the relationship between fiber content and beam bending strength by introducing a influence coefficient of fiber content.

(3) Increasing the content of SMA fibers can utilize the hysteresis energy dissipation characteristics of more fibers, thereby effectively improving the energy dissipation capacity of ECC beams. However, when the volume content of SMA fibers exceeds 0.6%, the fiber clustering will occur, resulting in the energy dissipation capacity decreases with the increase in SMA fiber content.

(4) When the SMA fiber content is less than 0.6%, the self-recovery ability of the ECC beam specimen increases with the increase in fiber content. When the SMA content is more than 0.6%, the self-recovery ability of the specimen decreases with the increase in fiber content. The self-recovery ability of the beam specimen is strongest when the SMA fiber volume content is 0.6%, and when the loading deflection is larger than 2.4 mm, the self-recovery rate is always higher than 60%, with a maximum recovery deflection of 4.68 mm and a maximum recovery rate of 80%.

However, the fabrication process of the knotted end used in this study is relatively complex, which is not favorable for large-scale applications. Therefore, it is necessary to explore simpler end forms in subsequent studies. In addition, the optimal fiber content of 0.6% obtained in this study is only applicable to the specific conditions of this test, and the optimal fiber content under other conditions needs to be determined by further research.

## Figures and Tables

**Table 1:** Mixture weight proportion of the ECC specimens.

Type	Cement	Fly Ash	Silica Sand	Water	Water Reducer	PVA * (%)
PVA-ECC	1.0	2.4	0.36	0.26	0.0082	2.00

* Percentage of fiber content by volume.

**Table 2:** PVA fiber performance index table.

Type	Length/mm	Diameter/µm	Tensile Strength/MPa	Elasticity Modulus/GPa	Elongation/%
PVA	9	31	1500	42	6

**Table 3:** Composite beam specimen table.

Specimen Type	Specimen Name	SMAF Diameter/mm	SMAF Volume Content	Specimen Number
SMAF-ECC beam	F-Y-0.2	1.0 mm	0.2%	3
	F-Y-0.3		0.3%	
	F-Y-0.4		0.4%	
	F-Y-0.5		0.5%	
	F-Y-0.6		0.6%	
	F-Y-0.7		0.7%	
	F-Y-0.8		0.8%	
	F-Y-0.9		0.9%	
	F-Y-1.0		1.0%	
ECC beam	E0	—	0	

(Note: In the specimen code F-Y-*, F represents the SMA fiber-reinforced beam, Y represents the knot-type end, and * represents the fiber content; E0 is the control specimen without SMA fibers.)

**Table 4:** Table of peak load and crack width.

Specimen Name	SMAF Volume Content	Peak Load/kN	Lifting Rate of Peak Load Relative to Comparison Specimen	Maximum Crack Width/mm	Residual Crack Width after Unloading/mm
Y-0.2	0.2%	2.54 kN	6.72%	6.1	2.4
F-Y-0.3	0.3%	2.69 kN	13.03%	5.2	2.3
F-Y-0.4	0.4%	2.81 kN	18.06%	6.5	2.3
F-Y-0.5	0.5%	2.69 kN	23.52%	5.8	2.0
F-Y-0.6	0.6%	3.53 kN	48.31%	5.7	1.2
F-Y-0.7	0.7%	3.10 kN	30.25%	6.2	1.8
F-Y-0.8	0.8%	2.91 kN	22.27%	5.8	2.0
F-Y-0.9	0.9%	2.83 kN	18.91%	5.8	2.0
F-Y-1.0	1.0%	2.63 kN	10.51%	5.6	2.2

**Table 5:** Bending strength table of specimen.

Specimen Name	SMAF Volume Content	Bending Strength/MPa
F-Y-0.2	0.2%	4.76
F-Y-0.3	0.3%	5.04
F-Y-0.4	0.4%	5.26
F-Y-0.5	0.5%	5.51
F-Y-0.6	0.6%	6.61
F-Y-0.7	0.7%	5.81
F-Y-0,8	0.8%	5.45
F-Y-0.9	0.9%	5.30
F-Y-1.0	1.0%	4.93
E0	0	4.46

**Table 6:** Calculation table of average deflection self-recovery rate of composite beams.

Specimen Name	SMAF Volume Content	Load Deflection/mm	Final Unloading Deflection/mm	Recovery of Deflection/mm	Deflection Recovery Rate/%	Standard Deviation
E0	0%	6	5.06	0.94	15.67	0.13
F-Y-0.2	0.2%		2.33	3.67	61.17	0.03
F-Y-0.3	0.3%		2.28	3.72	62.00	0.21
F-Y-0.4	0.4%		1.96	4.04	67.33	0.07
F-Y-0.5	0.5%		1.73	4.27	71.17	0.02
F-Y-0.6	0.6%		1.32	4.68	78.00	0.09
F-Y-0.7	0.7%		1.72	4.28	71.33	0.02
F-Y-0.8	0.8%		1.76	4.24	70.67	0.02
F-Y-0.9	0.9%		1.78	4.22	70.33	0.02
F-Y-1.0	1.0%		1.87	4.13	68.83	0.04

## Data Availability

Not applicable.
